# Extension for Prevention: A Study of Conditions in the Mouth

**Published:** 1904-11-15

**Authors:** Frederick B. Noyes

**Affiliations:** Chicago, Ills.


### EXTENSION FOR PREVENTION: A STUDY OF CONDITIONS IN THE MOUTH.

BY FREDERICK B. NOYES, B.A., D.D.S., CHICAGO, ILLS.

When, in 1891, Dr. Black first made use of the phrase which has attracted so much attention, it was as a result of his close observation of the conditions and positions of beginning caries of the enamel, and throughout the bitter opposition and in spite of quibbling sophistry which has sometimes been waged against the idea, he has maintained the same simple position, "Study the positions and conditions of beginning decay of the enamel, and plan and execute filling operations with reference to them." This constitutes extension for prevention.

### DETERMINING FACTORS IN "EXTENSION."

It is my purpose to confine myself to a study of the appearances which are the determining factors in extension. In the beginning it may be well to say that I am speaking of extension for prevention, not extension for convenience, extension for retention, or extension for contention.

The earliest observers of caries of the teeth noticed that decay begins at points upon the surfaces of the teeth, the tissue being affected by acids produced at the point of attack. The philosophy of filling, therefore, is that if the point of attack is cut out and replaced by an indestructible substance, the tooth is less liable to attack in the future.

Here we have the whole argument for and against extension for prevention. Those who oppose the idea take the ground that if the portion of the tooth which has been disintegrated be removed and replaced by indestructible material the tooth is no more liable to attack in the future than it would be if it were perfect in structure; and we certainly would not think of cutting into a perfect tooth and making a filling because it was possible that it might decay in that place some day.

On the other hand, extension for prevention is really a carrying of the original reasoning a little farther. While it is true that the teeth are attacked at points, the area of the attack differs greatly in different instances, and the intensity of the attack is always greatest at the central portion of the area. When, therefore, a cavity has been produced at the center of the area there can be no logic in advising the replacement of the lost portion of the tissue without removing the portions which are already showing signs of attack by the process.

The problem then becomes the recognition of the conditions which determine the size and shape of the areas of the tooth-surface which are liable to the attacks of caries. In this study there are many things to be taken into consideration, all of which are greatly modified by what may be called systemic susceptibility and immunity. Of these perhaps the first in importance is the manner of the attack and disintegration of the enamel.

### ACTION OF CARIES IN THE ENAMEL.

We can recognize two very distinct positions of attack in the enamel which present very characteristic appearances and manner of progress. First, attacks beginning in natural defects of structure, such as fissures and pits; second, in portions of smooth surfaces which are so protected from the friction of mastication as to afford lodgments for the gelatinous plaques which confine the acid products of microbic action.

We may consider the occlusal pits and fissures as typical of the first class, for whether on occlusal or axial surfaces, these defects are in positions which would be kept cleaned and polished if it were not for the rough and imperfect structure of the surface. Even with such defects we may have a degree of immunity which will prevent the attack on the tissue. When caries occurs in these defects, the opening of the pit or fissure is kept constantly cleansed and the acids formed are removed so rapidly that they do not attack the tissue; but in the depth of the crevice they are less rapidly removed, the opening often being blocked by foreign materials forced into it, and they are neutralized by the inorganic salts of the enamel, which they dissolve. In this action of acid upon the enamel we find that the cementing substance between the rods is first attacked, injuring the structure to a considerable depth. We find, therefore, that the destruction of tissue progresses most rapidly at the bottom of the pit or fissure, spreading laterally as well as toward the dento-enamel junction, and a considerable area of disintegrated tissue may be produced, or an actual cavity, without increasing the size of the original defect at the surface. This manner of spreading is characteristic of attacks beginning in natural defects. As soon as the dento-enamel junction is reached the destruction progresses more rapidly in the dentin at that point, which may lead to the entire undermining of the enamel without forming an opening on the surface which will attract any more attention than the original slightly-imperfect fissure.

The points of attack upon smooth surfaces bear no relation to the character of the tissue, the most perfectly organized apparently being as subject to destruction as the most imperfect, but are determined by the conditions in which the surfaces are placed and the character of the secretions which bathe them. In these points there is usually a center which represents the most favorable condition, and at this point the attack is most rapid and most penetrating, fading out in intensity toward the borders of the area to points which are constantly cleansed and which constitute areas of relative immunity. This produces an appearance in the tissue the reverse of that presented in attacks beginning in natural defects. In studying these areas of attack I have been surprised at the extent and the depth to which the tissue was affected without producing an opening which would be detected by an instrument. The cementing substance seems to be removed from between the rods, but they have not yet crumbled to pieces. Such spots in enamel will cut like chalk, and in grinding sections the tissue will usually crumble to pieces. These areas look white by reflected light or as seen on the tooth, if the attack is recent, because the light is refracted and diffused in passing through the rods and the surrounding air-spaces. If the conditions have changed and a state of immunity has occurred which has inhibited the action of the micro-organisms, the spaces in the tissue may be filled by foreign materials which become dark in color, and the white actively-growing spot becomes a stationary dark brown or black spot. It not infrequently happens that such superficial attacks are arrested by the coming of immunity before the tissue has been penetrated.

The beginnings of caries on smooth surfaces, then, are characterized by more or less broad areas of superficial disintegration, penetrating in a point corresponding to the point of greatest intensity, until the dentin is reached. The appearances can be well shown in sections through beginning decay.

### EXTENSION AS APPLIED TO NATURAL DEFECTS AND TO CAVITIES ON SMOOTH SURFACES.

In the paper in 1891, Dr. Black spoke of extension as applied to natural defects as generally recognized and practiced by the careful operator in the profession at that time. Still, it may be well to recall the conditions as shown by the microscope in grooves and fissures, as an illustration of the conditions which make this extension necessary. If we take teeth showing fairly well-closed grooves, on the occlusal surface and make sections through them, the imperfection in the union of the enamel plates will be surprising. If decay has reached the dentin at one point in the fissure, it must be cut out to the end of the fissure, or such defects will be left along the side of the filling material where the filling stops—often reaching half or two-thirds the distance to the dento-enamel junction. Such a condition may stand, if there be a systemic immunity in the mouth, but if a condition of liability occur, there is a natural opportunity left for the recurrence of attack.

These conditions, then, lead to the general statement that grooves and fissures should be cut out to their ends, or until a smooth margin can be formed. When this is not done and a defect is left, forming one of the boundaries of the filling, and if caries occur, it begins in the depth of the defect, and may not show upon the surface until the enamel begins to cave in. For this reason such fillings are very deceptive, and we can recall many cases from practice where we may have hesitated about removing an old filling, the defect scarcely seeming to justify it, but on removal the entire filling is found undermined and there is extensive decay in the dentin. If neglected, the whole crown might have been destroyed before the enamel would have been caved in. In these positions the reason for extension is entirely because of the imperfect structure, for there would be no beginning of caries if the surface were smooth enough to prevent lodgment and retention of decomposing food.

It is the second class of attacks, those beginning upon smooth surfaces, which specially demand our attention under the head of "Extension for Prevention."

Of the smooth surfaces the buccal or labial and the approximal are the ones which are subject to attack, caries on the lingual surface, except in structural defects, being very rare and occurring in exceptional conditions.

### AREAS OF IMMUNITY AND LIABILITY.

Before studying the appearances of superficial decay on the axial surfaces, and the conditions which modify the position and shape of the areas of attack, I want to call attention to the importance of the angles of the axial surfaces as the areas of greatest immunity, that is, the area stretching from occlusal to gingival at the union of the mesial and buccal, distal and buccal, distal and lingual, and mesial and lingual surfaces. Dr. Black called attention very strongly to these areas, as those upon which caries least often occur, in a paper read in February, at St. Paul. He said that in the examination of over eight thousand mouths of those under thirty years of age, there were but five cases where caries beginning on one surface had spread superficially across the angle on to the adjoining surface. In regard to this, he said: "Hence, as the lines of enamel margins approach these angles of the teeth they approach lines of safety. Whenever they overstep these lines, they have overstepped the lines of safety and have approached areas of increasing danger; this is especially true of all buccal and labial surfaces. . . . Therefore any cutting over these lines on the labial surfaces of incisors, or the buccal surfaces of bicuspids and molars, making fillings that are in bad taste from the esthetic point of view, are oversteppings of the rules of 'extension for prevention.'" This is intended as a warning to those who are inclined to overstep all bounds in extension.

The second area of immunity to be constantly remembered is the portion of the tissue covered by healthy gum. These two are the most positive regions of safety and even in cases of the greatest liability can usually be counted upon, provided the fillings are properly made and finished, and the patient can be made to properly chew food. They are of the greatest importance and must be constantly remembered in treating caries on all axial surfaces.

On the buccal surfaces of molars and bicuspids the area of liability is bounded on the mesial and distal by the axial line angles, on the gingival by the gum margin, and on the occlusal by the point of greatest convexity of the surface, or the point from which food in passing over the surface jumps from the surface of the tooth to the surface of the gum. The point of greatest intensity is usually about the middle of the surface mesio-distally, and the extent will depend upon the degree of liability, which is judged by the appearance of the surface and the general conditions—which are the indications as to the degree of the extension necessary.

### SYSTEMATIC TREATMENT OF CARIES.

The first step in the treatment of caries on any surface is the cleaning of that surface and the observation of the nature and extent of the superficial attack, on the character of which depends the extent of cutting. This must be judged not only in the light of the individual case, but of the operator's entire experience, and in this we must not neglect to consider the changes which occur in the areas of liability at different times. For instance, on the buccal surface of the lower molars in patients of thirteen or fourteen years of age, we very often find an area of corrosion just occlusally from the gum margin, with a point of greatest intensity lying about the mesio-distal center of the buccal surface and often corresponding in position to the buccal pit, at which point the enamel is often penetrated. In preparing such a cavity for filling we may not be called upon to cut out the entire extent of superficial attack as indicated by the white lines stretching away to mesial and distal. For as soon as this tooth reaches its full length these lines will be carried into areas of comparative immunity and the disintegration of tissue will stop. As the tooth erupts and the gum recedes, then the area of liability is carried farther to the gingival.

On the labial surface of incisors and cuspids we have the same general conditions. When caries occurs it is either in a natural defect or just occlusally from the gum margin, extending in a crescent form, following the curve of the gum margin, the occlusal boundary of the area being the last point at which food exerts friction upon the tooth in mastication, or at which the lip rubs over the surface with sufficient force to keep it clean. The point of greatest intensity is generally just under the line of greatest convexity of the surface, which is usually the center of the surface from mesial to distal in cuspids, but is often a little to mesial or distal of the central line in incisors. This point of greatest intensity is often modified by rotations of the teeth which cause food to pass over the surfaces in a slightly-unusual manner.

### IN YOUNG PEOPLE.

In young people of great susceptibility we sometimes find labial cavities in the middle third of the surface, which appear while the tooth is in the process of eruption, while the gingival third is still covered by the gum. These call for great vigilance but not great extension, for the area of liability will leave the margin in safe positions when the tooth has reached its full eruption. Unless these cases are watched very carefully, the tissue will be destroyed as the area of liability passes over it. Cavities in the gingival third, however, must be extended so as to leave the margin permanently under the gum margin, and must be so finished that the tissue will remain healthy and covering it, for if it recedes so as to leave the margin exposed, a new area of liability will appear between the margin of the filling and the gum. The importance of perfect finishing and polishing cannot be overestimated in these positions, and it is often very difficult to accomplish.

On the approximal surfaces the conditions are complicated by the presence of the approximating tooth. These surfaces are best considered in two groups—the incisors and canines in one, the molars and bicuspids in the other.

In the incisors and cuspids the area of liability is roughly triangular, extending from the contact point toward the labio-gingival angle in a fairly straight line, from the contact point to the linguo-gingival angle in the same way, and bounded gingivally by a curved line just occlusally from the gum margin. The point of greatest intensity is toward the gingival from the contact point. The size of the area depends upon the age and the degree of liability. In general the area increases with age and the moving of the gum margin gingivally and the intensity in the area decreases. In all approximal surfaces the typical areas may be modified by abnormal or exceptional relations of the approximating teeth. The difference in intensity of attack between the point toward the gingival from the contact and the rest of the area is usually very considerable, and much greater than in the case of buccal and labial areas. Penetration, therefore, almost always occurs at this point, but this may be located very close to the incisal edge, or it may be in the occlusal half of the middle third of the surface. When these cases present for treatment the area of broken-down enamel is almost always nearly circular, but if the surface of the tooth is cleaned and polished, whitened areas of enamel will be seen extending to labio-gingival and linguo-gingival from the border of the cavity. These areas should be included in the cavity, or the surface attack continuing in these directions will lead to the formation of a new cavity in these portions at the margin of the old one. It must be remembered, too, that the area will extend farther to the gingival in the future; so that, especially in young peoplee the gingival wall must be carried beyond the point of surfac, attack toward the gingival. The gingival wall should be made flat, at right angles to the axis of the tooth, extending from the farthest point of attack at the labio-gingival to the similar point at the linguo-gingival, which will carry it well under the gum margin across the approximal surface. In building the filling it is often necessary to modify the contour in such a way as to bring the contact point a little to the gingival of the incisal margin, and avoid having the incisal margin pass through the contact point. Extension for prevention does not call for carrying the labial margin around the angle on to the labial surface, bringing the gold into view.

The conditions on the approximal surfaces of the bicuspids and molars are the most complicated and, at the same time, the most interesting, and in these positions we most often see disastrous results from a neglect to comply with the conditions. The area of liability in bicuspids and molars is roughly oval in form. The occlusal border passes through the contact point horizontally and out into the embrasures in a curve, running toward the bucco-gingival and linguo-gingival angles of the surfaces in a curve and following the outline of the gum margin across the approximal surface. The bucco-lingual diameter of the area is usually greater than the occluso-gingival, and the points of greatest intensity is represented by a small oval just to the gingival of the contact point. It is at this point that penetration occurs oftenest in young people. The size of the area of liability is increased in proportion as the area of contact is broad and as the teeth are short from occlusal to gingival, because of the greater tendency to the retention of food and the protection of microbic plaques around the area of contact. The treatment of these conditions requires the increase of the mesio-distal diameter of the tooth, increasing the inter-proximal space, narrowing of the contact point and increasing the width of the embrasure. From youth to middle age the extent of the area increases bucco-gingivally and linguo-gingivally, though the intensity decreases, if the form of the contact and the inter-proximal space be maintained. This requires an extension gingivally of the gingival wall beyond the limits of superficial attack for young people, which consists in a squaring out of the gingival corners, following and passing the indications of superficial attack.

In the treatment of caries of approximal surfaces of bicuspids and molars, "extension for prevention" is useless unless the proper form of contact and inter-proximal space be restored, and they had better not be extended unless the fillings are properly made and properly finished and polished.

### IN PERSONS OF MIDDLE AGE AND OLDER.

In persons of middle age and older there is another form of decay on approximal surfaces caused by the flattening of approximal contacts by the wear of badly-contoured fillings in the adjoining tooth. In this class of cavities decay occurs much farther to the gingival, often at the gingival line, producing very ugly cavities. These call, not for extension for prevention, but for restoration of self-cleansing contacts. This can only be done by slow or repeated separation and the building of properly-contoured fillings.

In the consideration of this subject, caries is treated as primarily a disease of youth. It does not consider the forms of decay usually associated with cachectic conditions in old people.

In closing I simply want to say that "extension for prevention" should never be followed empirically, by rules to cut to here or to cut to there, but based upon a study of the conditions of beginning decay of the enamel, especially as manifested by superficial corrosion of the surfaces of the teeth, and the cavity margins placed in positions and under conditions that will be least liable to recurrence of decay. We cannot do better than to take the example of the father of the expression, who takes just pride in the number of mouths of which he can say, "I cared for them from youth through middle age, and they never lost a tooth and never lost a filling." To accomplish such things requires careful study of conditions in the mouth, skillful manipulation and a sympathy of heart—qualities possessed by few to a higher degree than by the author of "extension for prevention." Dr. G. V. Black.—October *Cosmos*.

